# Clinical effect and safety evaluation of different dosage of Rituximab combined with Cyclophosphamide in treatment of refractory immune Thrombocytopenia

**DOI:** 10.12669/pjms.36.2.1168

**Published:** 2020

**Authors:** Zhibin Wang, Yu Ren, Mingwei Li, Weibo Huang, Haiying Yao

**Affiliations:** 1 Zhibin Wang, Department of Hematology, Baoding First Central Hospital, Hebei, Baoding 071000, P. R. China; 2 Yu Ren, Department of Hematology, Baoding First Central Hospital, Hebei, Baoding 071000, P. R. China; 3 Mingwei Li, Department of Hematology, Baoding First Central Hospital, Hebei, Baoding 071000, P. R. China; 4 Weibo Huang, Department of Hematology, Baoding First Central Hospital, Hebei, Baoding 071000, P. R. China; 5 Haiying Yao, Department of Hematology, Baoding First Central Hospital, Hebei, Baoding 071000, P. R. China

**Keywords:** Coagulation function, Cyclophosphamide, Lymphocyte, Refractory immune thrombocytopenic purpura, Rituximab, Untoward effect

## Abstract

**Objective:**

To discuss clinical effect of different dosage of rituximab combined with cyclophosphamide in treatment of refractory immune thrombocytopenia (rITP).

**Method:**

This study was conducted at Department of Hematopathology in XX Hospital from January 2016 to January 2018. In this study. Seventy-eight patients with rITP were selected as the objects, divided into observation group (39 cases) and control group (39 cases) according to random number table. Patients in the control group were treated with conventional rituximab and cyclophosphamide, while the observation group received low-dose rituximab. The same amount of cyclophosphamide was used in the two groups. The statistics of clinical effect, recurrence rate, untoward effect and Laboratory inspection of both groups were made before and after the treatment.

**Results:**

Compared with the control group, the total occurrence rate of side effects in the observation group decreased significantly; the level of IgM and CD20^+^ in the observation group also decreased significantly, while. The level of IgA, IgG, CD3+ and CD4+ rose significantly (P<0.05). The differences in the level of Th1, TNF-a, IL-18 and Sc5b-9 had statistical significance before and after the treatment (P<0.05).

**Conclusion:**

Rituximab combined with cyclophosphamide has the definite curative effect on rITP. The small dosage of rituximab combined with cyclophosphamide has higher clinical safety in the treatment.

## INTRODUCTION

Primary immune thrombocytopenia (ITP), also called idiopathic thrombocytopenic purpura, is a common hemorrhagic disease.^1^,^2^ At present, the pathogenesis of ITP is not clear. It is generally believed that, ITP is related to patients’ body immunity dysfunction.^3^ About 70% of patients choose glucocorticoid, immune globulin, splenectomy and immunosuppressor^4^, where 20~30% of patients with ITP turn into rITP due to treatment failure or reoccurrence.^5^ The patients with rITP generally lack sensitivity to hormonotherapy. Even if the high dosage of conventional medicine is used, it is still difficult to gain certain curative effect.^6^,^7^ Therefore, to seek an effective treatment plan for the patients with rITP is the concern of every clinicians. Rituximab as a kind of human-rat chimeric monoclonal antibody has specific recognition ability and can destroy B lymphocyte.^8^ It has significant curative effect on treatment of rITP. In this study, the curative effect of conventional dosage and small dosage of rituximab combined with cyclophosphamide in the treatment of rITP is mainly discussed as follows:

## METHODS

Seventy-eight patients with rITP who were received and treated by Department of Hematopathology in XX Hospital from January 2016 to January 2018 were chosen as the objects of study, including 14 male patients and 64 female patients. Their age was between 16 and 68, with the median age of 35.5.

### Inclusion criteria:

1. All patients conformed to diagnostic criteria of rITP;
2. Disease course ≥12m;
3. Platelet count (PLT) <30×10^9^/L or hemorrhage;
4. Normal number of megakaryocytes or the increase of megakaryocytes found in bone marrow aspiration, accompanied with dysmaturity.

### Exclusion criteria:

1. Children, pregnant women and lactating women;
2. Those sensitive to rituximab or rat protein;
3. Persons infected by hepatitis B, hepatitis C virus or HIV;
4. With other autoimmune diseases such as systemic lupus erythematosus, lymphatic proliferative disease, congenital or hereditary thrombocytopenia;
5. Heart, liver and kidney dysfunction. The patients were classified into observation group and control group according to random number table. Each group included 39 patients. All patients had hemorrhage to different degrees.

Both groups had no statistical significance in terms of gender, age, disease course and platelet count (P>0.05). Both groups had comparability, as shown in Table-I. This study gained patients’ informed consent and the approval of Ethics Committee dated September 20, 2018. All patients signed informed consent Form.

**Table-I:** Statistics of general conditions of rITP patients in both groups.

Group	No.	Gender		Age	Disease course	Platelet count

		Male	Female	(Year)	(year)	(10^9^/L)
Observation group	39	14	25	34.1±4.1	4.9±1.1	12.0±1.5
Control group	39	14	25	33.3±4.2	5.0±4.2	12.7±1.4
t/X^2^		--	0.695	0.362	1.655	
P		>0.05		>0.05	>0.05	>0.05

Intravenous drip of conventional dosage of rituximab (375mg/m^2^) (subpackage approval: GYZZ J20170002, subpackage batch No. of imported medicine: S20170002; S20160029, subpackage enterprise: Shanghai Roche Pharmaceuticals Ltd.) was given for the control group once per week, and the drip duration each time was above 1h. Besides, intravenous drip of 0.8g cyclophosphamide (produced by Jiangsu Shengdi Pharmaceutical Co. Ltd., batch No.: 18022825) was given for the control group once per week, and 2mg/(kg.d) cyclophosphamide was taken sequentially. Intravenous drip of small dosage of rituximab (100mg/m^2^) was given for the observation group once per week. The same amount of cyclophosphamide was used in the two groups. The treatment duration for both groups was four weeks. To avoid anaphylaxis, separate drip of 5mg dexamethasone (produced by Tianjin Jinyao Group Co., Ltd., batch No.: 1706252) was given before the drip.

### Laboratory examination

Blood routine and four coagulation items were examined twice weekly during the treatment. In case of hemorrhagic tendency, blood routine and four coagulation items should be examined immediately. Before the treatment and four weeks after the treatment, flow cytometry was applied to detect peripheral blood lymphocyte and regulatory T cells. Quantitative determination of serum immunoglobulin was conducted with immunoturbidimetry. Enzyme linked immunosorbent assay (ELISA) was adopted to test patients’ blood biochemistry level.

### Observation indexes:

1. Clinical effect and recurrence rate of both groups were compared. Therapeutic evaluation criteria are as follows.^9^ Complete response: blood platelets rise to 100×10^9^/L (Test twice more than seven days apart) and there is almost no hemorrhage. Response: blood platelets rise to 30×10^9^/L (Test twice more than seven days apart) and there is almost no hemorrhage. No response: blood platelets below 30×10^9^/L or Less than two times of that before treatment (It must be measured twice more than one day apart), and there is no hemorrhage. The cases with complete response and response total to be effective. The computational formula of total effective rate is [(cases with complete response and response)/total cases]. Recurrence: after the effective treatment, the platelet counts declines below 30×10^9^/L or hemorrhage appear.
2. PLT, HGB content, PT and aPTT of both groups were compared before and after the treatment.
3. The changes in the level of serum immune globulins IgA, IgM and IgG, and the changes in the level of lymphocytes CD3^+^, CD4^+^ and CD20^+^ were compared.
4. The level of Th1, TNF-a, IL-18 and Sc5b-9 were compared.
5. The statistics of total occurrence rate of side effects was conducted for both groups.

### Ethical approval

The study was approved by the Institutional Ethics Committee of our hospitals, and written informed consent was obtained from all participants.

### Statistical method SPSS 22.0 statistical package was applied for data analysis

Enumeration data were expressed with %, and measurement data were expressed with (x±s). Chi-square test and *t* value test were used for inter-group comparison, while one-way analysis of variance was adopted for intra-group comparison. P<0.05 means the difference has statistical significance.

## RESULTS

The efficacy rate of observation group was 53.85%, and the effective rate of control group was 64.10%. The difference had no statistical significance (P>0.05). The recurrence rate of observation group was 5.13%, while the figure was 5.13% for the control group. The difference had no statistical significance (P>0.05). Two cases relapsed respectively in both groups, as shown in Table-II.

**Table-II:** Clinical effect comparison for rITP patients in both groups [n/(%)] Comparison of coagulation indicators before and after treatment.

Group	No.	Significant effect	Good effect	Progress	No effect	Total effective rate	Recurrence rate
Observation group	39	13(33.3)	8(20.5)	11(28.2)	7(17.9)	21(53.85)	2(5.13)
Control group	39	16(41.0)	9(23.1)	8(20.5)	6(15.4)	25(64.10)	2(5.13)
X^2^						0.845	--
P						>0.05	>0.05

PLT level of both groups improved significantly after the treatment (P<0.05). The comparison of HGB level had no statistical significance (P>0.05). The level of PT and aPTT declined significantly, compared with pre-treatment (P<0.05). Inter-group comparison of various indexes had no statistical significance (P>0.05), as shown in Table-III.

**Table-III:** Comparison of coagulation function and HGB before and after treatment (x±s) (n=39)

Group	PLT(S)		HGB (g/L)		PT(10^9^/L)		aPTT(S)	

	Pre- treatment	Post- treatment	Pre- treatment	Post- treatment	Pre- treatment	Post- treatment	Pre- treatment	Post- treatment
Observation group	12.29±0.55	85.21±4.07*	8.97±0.63	10.58±0.94	18.64±1.31	17.18±1.30*	41.30±2.62	35.25±2.43*
Control group	12.65±0.84	87.79±3.28*	9.15±0.58	10.92±0.98	19.28±1.18	16.74±1.37*	42.37±3.00	36.09±2.28*
t	-1.440	-1.982	-0.855	-0.997	-1.449	0.914	-1.074	-1.012
P	>0.05	>0.05	>0.05	>0.05	>0.05	>0.05	>0.05	>0.05

***Note:*** comparison before and after the treatment: P*<0.05.

The level of IgM and CD20^+^ in the observation group was significantly lower than that of the control group. The level of IgA, IgG, CD3+ and CD4+ in the observation group was significantly higher than that of the control group (P<0.05), as shown in Table-IV.

**Table-IV:** Comparison of indicators of serum immune globulins and lymphocytes before and after the treatment (x±s) (n=39).

Group	IgA (g/L)		IgM (g/L)		IgG (g/L)	

	Pre-treatment	Post-treatment	Pre-treatment	Post-treatment	Pre-treatment	Post-treatment
Observation group	0.98±0.12	0.89±0.21*	1.65±0.25	1.72±0.32*	10.35±2.34	9.82±2.42*
Control group	0.97±0.11	0.42±0.09*	1.68±0.27	3.54±0.75*	10.32±2.25	4.62±1.04*
t	0.384	12.847	0.509	13.939	0.058	12.329
P	>0.05	<0.05	>0.05	<0.05	>0.05	<0.05
Group	CD3+ (10^9^/L)		CD4+ (10^9^/L)		CD20+(10^9^/L)	
	*Pre-treatment*	*Post-treatment*	*Pre-treatment*	*Post-treatment*	*Pre-treatment*	*Post-treatment*
Observation group	1.65±0.36	1.48±0.28*	0.98±0.23	0.81±0.29*	27.9±3.8	17.1±2.1*
Control group	1.64±0.38	1.18±0.29*	0.96±0.25	0.53±0.20*	28.0±3.6	16.3±2.0*
t	0.119	4.648	0.368	4.964	0.092	16.143
P	>0.05	<0.05	>0.05	<0.05	>0.05	<0.05

Note: comparison before and after the treatment: P*<0.05.

The changes in the level of serum Th1, TNF-a, IL-18 and Sc5b-9 before and after the treatment had no statistical significance (P>0.05). The inter-group comparison of various indexes had statistical significance (P<0.05), as shown in Table-V.

**Table-V:** Comparison of biochemical indicators before and after the treatment (x±s) (n=39)

Group	Tn1(%)		TNF-a (μg/L)		IL-18 (pg/mL)		Sc5b-9 (ng/mL)	

	Pre- treatment	Post- treatment	Pre- treatment	Post- treatment	Pre- treatment	Post- treatment	Pre- treatment	Post- treatment
Observation group	20.82±1.49	22.83±1.90*	72.63±10.35	62.07±8.52*	268.90±57.19	224.00±39.39*	677.05±102.71	516.93±99.39*
Control group	20.01±1.26	21.80±1.89*	77.62±8.57	63.86±9.41*	272.29±56.89	230.68±45.28*	675.90±95.90	538.15±95.78*
t	2.061	1.838	-1.486	0.700	-0.168	0.688	0.033	0.545
P	>0.05	>0.05	>0.05	>0.05	>0.05	>0.05	>0.05	>0.05

***Note:*** comparison before and after the treatment: P*<0.05.

Liver and kidney damage did not appear for both groups in the treatment period. Based on the statistics of urticarial, fever, shiver, pulmonary infection, dyspnea, alimentary tract hemorrhage and gastrointestinal reaction, the differences in the total occurrence rate of side effects had statistical significance (P<0.05), as shown in Table-VI.

**Table-VI:** Comparison of side effects [n/(%)] (n=39).

Group	Liver and kidney damage	Urticarial	Fever	Shiver	Pulmonary infection	Dyspnea	Alimentary tract hemorrhage	Gastrointestinal reaction	Total occurrence rate of side effects
Observation group	0	0	1(2.56)	1(2.56)	0	0	0	2(5.13)	4(10.26)
Control group	0	2(5.13)	2(5.13)	3(7.69)	1(2.56)	1(2.56)	0	2(5.13)	11(28.21)
X^2^									4.287
P									<0.05

## DISCUSSIONS

rITP is a kind of autoimmune diseases of the blood system. During the treatment of ITP, the most common medicine is glucocorticoid. However, some ITP patients are not sensitive to glucocorticoid or resist glucocorticoid. Thus, glucocorticoid treatment failed for some ITP patients, which might be influenced by heat shock protein in ITP patients’ peripheral blood mononuclear cells.^10^ The main research progress of rITP lies in the use of rituximab.^11^ CD20^+^ is an important antigen participating in signal transduction, and regulation of B cell growth and differentiation. Rituximab is human-rat chimeric CD20^+^ monoclonal antibody for CD20^+^ molecule on B lymphocyte surface, and it can inhibit B lymphocytes to generate antibody, and reduce the destruction of blood platelets.^12^ Therefore, rituximab is a common medicine to eliminate B cells.^13^ Rituximab as the second-line treatment medicine of ITP is recommended to treat rITP. The patients with ITP (good treatment effect with glucocorticoid) and secondary immune thrombocytopenia have good therapeutic response to rituximab.^14^ A study showed that, rITP patients treated with standard dosage of rituximab, and the effective rate of treatment could increase by 70%. Besides, patients’ platelet count rose obviously.^15^ The curative effect of standard dosage of rituximab in treatment of ITP can be basically affirmed.

Cyclophosphamide can inhibit cell proliferation, and has no specificity for antigen-sensitized lymphocytes. Cyclophosphamide plays the role of inhibition in cellular immunity and humoral immunity. At present, it has been widely applied in the treatment of autoimmunity and lymphatic systemic diseases.^16^ There has been no unified standard about the therapeutic dosage of rituximab for the adult ITP patients. Currently, standard treatment scheme of ITP with rituximab (375 mg/m^2^, once per week, use for consecutive 4w) stemmed from lymphoma treatment. But because the number of B cells of ITP patients is far fewer than that of lymphoma patients, after the treatment with standard dosage of rituximab, CD20 cells in peripheral blood almost disappear, which increases the infection chance.^17^ At present, the most important problem perplexing doctors and patients is how to control the blood platelet level of rITP patients who have no effect on hormone or depend on hormone. Therefore, the clinical effect of rituximab combined with cyclophosphamide in the treatment of rITP patients, and the optimal dosage of rituximab were investigated in this paper.

In this study, after rITP patients were treated by rituximab combined with cyclophosphamide, peripheral blood lymphocyte CD20^+^ was negative, which proves that rituximab gives play to the good effect of scavenging agent for B lymphocytes, and the decreasing function for ITP autoantibody, thus further relieving the destruction to blood platelets. Patients’ PLT and HGB level rose significantly compared with pre-treatment, while PT and aPTT level significantly declined, compared with pre-treatment. This indicates that, after the treatment with the scheme, platelet count, hemoglobin count and coagulation function of both groups recovered effectively, and could reach the satisfying short-term curative effect. The level of IgM and CD20^+^ in the observation group reduced significantly, compared with the control group. The level of IgA, IgG, CD3+ and CD4+ rose significantly, compared with the control group. This means that, after the treatment, immune system damage of the patients in the observation group is obviously lower than that in the control group. Standard dosage of rituximab may damage the functions of immune cells and normal B lymphocytes, and increases the potential risk of serious infection. Cell factors of both groups such as Th1, IL-18, TNF-α and sC5b-9 declined obviously, which indicates that rituximab combined with cyclophosphamide can improve patients’ immunologic disorder and has significant curative effect on rITP.

The treatment safety of rituximab combined with cyclophosphamide is positive. The occurrence rate of side effects in the observation group is obviously lower than that in the control group. This means the low dosage of rituximab is safer and more effective. In the treatment process, rituximab and cyclophosphamide had certain influence on patients’ peripheral blood leucocyte, liver and kidney functions. Traditional dosage of rituximab may cause patients’ humoral immunity is damaged for a long term, and even serious infection and reactivation of hepatitis B virus may be caused. To reduce the treatment risk, nucleoside meducines such as lamivudine may be applied preventively.^18^

## CONCLUSION

Small dosage of rituximab combined with cyclophosphamide can significantly improve clinical symptoms of rITP patients, and has good curative effect, without serious untoward effect. So, the treatment scheme has high safety. However, the medicine is expensive. Thus, the researchers tried to apply small dosage of rituximab combined with cyclophosphamide to treat rITP. It was found that, the curative effect was close to the standard dosage of rituximab combined with cyclophosphamide. So, this treatment scheme deserves to be promoted and encouraged.

### Authors’ contributions:

**ZW & HY** designed this study and significantly revised the manuscript.

**YR, ML & WH** performed this study and drafted the manuscript, are responsible for integrity of research
